# The role of job aids in supporting task sharing family planning services to community pharmacists and patent proprietary medicine vendors in Kaduna and Lagos, Nigeria

**DOI:** 10.1186/s12913-022-08360-0

**Published:** 2022-08-01

**Authors:** Sikiru Baruwa, Elizabeth Tobey, Emeka Okafor, Kayode Afolabi, Toyin O. Akomolafe, Innocent Ubuane, Jennifer Anyanti, Aparna Jain

**Affiliations:** 1 Population Council, House 4, No. 16 Mafemi Crescent, Off Solomon Lar Way, Utako, Abuja, Nigeria; 2 Population Council, Washington D.C., USA; 3 Society for Family Health, Abuja, Nigeria; 4 Federal Ministry of Health, Abuja, Nigeria

**Keywords:** Task shifting and task sharing, Knowledge retention, Community pharmacists, PPMVs, FP jobs aids, FP knowledge, Knowledge of injectable contraceptives, Knowledge of implant contraceptives

## Abstract

**Background:**

CPs and PPMVs are an important source of modern contraceptives in Nigeria, yet many lack the requisite knowledge and skills to capably provide these services. This skills gap might be addressed through targeted family planning (FP) training. This study measures family planning knowledge retention of CPs and PPMVs after receiving training in FP counseling and services in Kaduna and Lagos States, in Nigeria.

**Methods:**

In a quasi-experimental longitudinal design without a comparison group, 559 CPs and PPMVs who were enrolled in the IntegratE project between January and December 2019, completed a self-administered questionnaire to assess their knowledge related to the provision of FP counseling, and injectable and implant contraceptive services at three points in time: 1) before the training; 2) immediately after the training; and 3) 9-months after the training in Kaduna and Lagos states, Nigeria. Adjusted multivariate logistic regression analysis was used to assess the effect of provider characteristics and receipt of job aids on FP knowledge retention 9 months after the training. 95% confidence intervals and *p*-values were used to assess statistical significance.

**Results:**

Majority of study participants were females (60.3%) and between 30 and 49 years old (63.4%). The study revealed the importance of jobs aids as influence on knowledge retention. CPs and PPMVs who reported having the Balanced Counseling Strategy plus (BCS+) counseling cards, were more likely to retain knowledge (AOR: 2.92; 95% CI: 1.01–8.40, *p*-value = 0.05) at 9 months follow-up. Similarly, in terms of knowledge of injectable contraceptives, CPs and Tier 2 PPMVs who reported receiving the Medical Eligibility Criteria (MEC) Wheel were 2.1 times more likely to retain knowledge of injectable contraceptives 9-months later on (95% CI: 1.14–3.99, *p*-value = 0.02).

**Conclusion:**

Community Pharmacists and Proprietary Medicine Vendors had good retention of family planning knowledge, especially when combined with job aids. Training and providing them with job aids on FP will therefore support task shifting and task sharing on family planning services provision in Nigeria.

## Introduction

Despite many years of concerted efforts to improve family planning (FP) services and uptake by national governments and international organizations, unmet need for family planning remains high globally [1]. Four of the six countries with the highest unmet need for FP are in sub-Saharan Africa (SSA) and include Democratic Republic of the Congo (DRC), Uganda, Nigeria, and Kenya [2]. In Nigeria, 15% of all women of reproductive age (15–49) have an unmet need for FP [3]. The current prevalence rate for modern contraceptive use in Nigeria is appropriately 12% [3]. This translates to low levels of FP uptake and unwanted pregnancy and correlates with high maternal mortality ratio [4]. Many women who need family planning are not using contraception” [3]. Long-acting methods such as implants, and intrauterine devices (IUDs) are mainly available in public health centres while short acting reversible methods like emergency contraception, male condoms, and pills are primarily available from private chemists/patent medicine store [3]. Providing women with access to a full range of methods, including access to long-acting methods is important in ensuring they have the right to choose the method that is best suited to their needs.

About 41% of women in Nigeria who use a modern contraceptive method receive their method from the private sector [3]. Community pharmacists (CPs) and Patent Proprietary Medicine Vendors (PPMVs) are popular sources of contraception among both public and private sources with about 22% of modern contraceptive users reported receiving their last method from a PPMV, and 12% from a community pharmacy [3]. A CP is a trained pharmacist with full license to sell and buy prescription and non-prescription drugs. PPMVs are “persons without formal pharmacy training selling orthodox pharmaceutical products on a retail basis for profit” [5]. In 2005, the number of PPMVs in Nigeria was estimated to be 200,000, roughly 100 times greater than the number of registered pharmacies, and nearly four times the number of physicians [6]. CPs and PPMVs are both currently regulated by the Pharmacists Council of Nigeria (PCN).

The main reasons why women reported use of the private sector include widespread availability, consistent drug stocks, extended hours, personable interactions, and no separate fees for consultations [7, 8]. However, CPs and PPMVs have not received formal training to provide FP services and often lack the requisite knowledge and skills to competently provide these services. Previous studies have shown that while many PPMVs provide FP services, they do not have the required knowledge to do so [9, 10].

### Task shifting and sharing policy (TSTS)

Based on the realization of severe health care worker shortage globally especially in sub-Sahara Africa, the World Health Organization (WHO) proposed task shifting to improve access to health care services [11]. Task shifting involves shifting some tasks from one cadre to another, for example from doctors to nurses, midwives, or community health workers [11]. In Sub-Sahara Africa, the shortage of health work force is further heightened by a high burden of diseases; migration of trained health workers; lack of enabling work environment, and low staff morale [12, 13]. The WHO provided global guidance on the process of TSTS implementation, which includes guidance on consultation, situation analysis, national endorsement, and regulatory framework. WHO guideline also specifies elements of quality assurance such as standardized training, supportive supervision, and certification and assessment, necessary to ensure quality of care [11].

To increase access to health services in Nigeria, the Federal Ministry of Health (FMOH) developed a TSTS Policy Guidelines in 2014 [14]. The TSTS policy focuses on key priority areas such as Reproductive Health, Maternal and Child Health (RMNCH), family planning, HIV (human immunodeficiency virus), tuberculosis, malaria and other communicable and non-communicable diseases in the essential health services package [14].

As part of the Federal Ministry of Health (FMoH) commitments to increasing access to high-quality FP services, efforts are being made to include CPs and PPMVs in the TSTS policy. The TSTS policy specifies that essential health care services of some family planning services can be shared with lower cadre health workers such as the Community Health Extension Workers [14]. Regarding FP, task sharing is commonly practiced in the public health sector to community healthcare workers [15, 16] but not implemented often in the private sector with CPs and PPMVs despite it being considered a high impact practice in FP [17]. Literature has revealed that task shifting some of the family planning services to other cadres may be cost effective, increase access to and availability of maternal and reproductive health services without compromising standard of service delivery [18]. However, task shifting can only be successful when supported by training, services restructuring, mentoring, supervision, and ongoing support from existing health system structures [19–21].

Training is a key strategy to build capacity of CPs and PPMVs to offer quality FP services, although some studies have shown that only about 30% of CPs and PPMVs received any training on FP counseling or provision of services [22, 23]. Most trainings are targeted to public-sector providers [24] and not CPs and PPMVs. Where opportunities were available to train CPs and PPMVs, as business owners they were concerned about forgoing profits when participating in trainings. Trainings that address this barrier may better attract private sector participants and retain their participation throughout the training.

A systematic review [25] of the effect of FP trainings on service delivery in the public sector showed that FP trainings can positively influence provider knowledge, the quality of counseling and services rendered. One of these studies reviewed [26] found that FP training is positive in enhancing providers’ average knowledge scores of side effects and warning signs for oral contraceptives and IUDs. They also found that providers not only increased their knowledge of contraception but also offered better quality of care to their clients. Another study in Nigeria reported a positive correlation between training and quality of FP counselling where trained nurses performed better in interpersonal relations, information giving, counseling, and mechanisms for encouraging continuity [27]. A study in the Philippines found that a FP training intervention positively improved providers’ knowledge and quality of care received by clients [28]. In Ghana, a study found that trained counsellor provided more detailed and full information about all available contraception [29].

In the private sector, three studies have shown that FP trainings can improve provider knowledge and quality of services. A study in Gujarat, India, showed that after training in FP methods, private medical practitioners FP knowledge improved, and this resulted in better care for their clients [30]. Another study in Nepal found positive improvements in client satisfaction at intervention clinics as compared to the control [31]. In Nigeria, previous studies reveal that PPMVs’ knowledge of injectable contraceptives increased with training [32, 33] and PPMVs’ FP clients are also generally satisfied with the services received [10, 32]. However, one study found no impact of training on provider behavior. A Study in Peru that assessed the effect of quality-of-care improvements after provider training on the job aids, found weak connection between training providers on FP job aids training and client’s outcomes [34].

Our study builds on this body of literature and explores the effect of FP trainings on CPs and PPMVs knowledge retention. The study also looks at the role of job aids in supporting knowledge retention over time.

### The IntegratE project

The IntegratE Project, supported by Bill & Melinda Gates foundation and Merck Sharp & Dohme (MSD) for mothers was implemented by a consortium of partners led by Society for Family Health in Nigeria. Other partners include Marie Stopes International of Nigeria, PharmAccess Foundation, Planned Parenthood Federation of Nigeria, and the Population Council. The project adopted a tiered accreditation approach where PPMVs were categorized into three tiers based on their health qualifications that determines the type of FP training and services provided. Tier 1 providers have no health qualifications, and offer FP counseling and basic FP services, while Tier 2 and 3 providers have health qualifications and provide additional FP services including long acting reversible contraceptive (LARC) methods. Each tier was authorized to practice FP services consistent with their qualifications (see Table 1). Included in the study are CPs as a cadre outside the PPMV categorization and were eligible to provide an expanded range of FP services once trained. Marie Stopes International, one of the IntegratE consortium members trained all providers in FP counseling using the balanced counseling strategy plus (BCS+) toolkit for three days. Sessions focused on (a) different contraceptive methods available in Nigeria; (b) instruction and practice sessions to offer client-centered FP counseling using BCS+; and (c) instruction on how and when to refer clients for FP to other sources of care based on client’s medical eligibility.

**Table 1:** Tiered Accreditation Training

	ELIGIBILITY CRITERIA	SCOPE OF WORK (Pharmaceutical care and Service Delivery)
**Tier 1 PPMVs (i.e. PPMVs lacking health qualifications and any training**	1. Ability to read and write. 2. Attainment of 21 years of age. 3. Qualified personnel will include those who attempted to obtain a First School Leaving Certificate. 4. Also, in this category are Bachelor’s degree in biological sciences or any other disciplines (outside of health related disciplines	1. Sell OTC and manage common illnesses. 2. FP services will include condoms, cycle beads, emergency contraceptives, refill of pills but not initiation, counselling, and referrals. 3. However, persons who have training on the use of malaria rapid diagnostic test-kits (m RDT) may use the test kits to test patients for malaria.
**Tier 2 PPMVs (i.e. Health Qualified PPMVs):**	1. Must fulfil the tier 1 eligibility criteria. 2. In addition, must possess degree in Health discipline as recognized by the Pharmacists Council of Nigeria 3. They could be Nurses, Midwives, CHEWs, CHOs	1. Provide select PHC services in line with the **Task shifting and Task sharing policy.** 2. Provide tier 1 services as well as use Rapid Diagnostic Test kits (RDTs), administer amoxicillin DT, conduct HIV Self Testing, sell self-injecting contraceptives and refer patients to PHCs and higher level facilities for nutrition counselling and treatment of any other common ailment. 3. They will also be allowed to initiate and administer LARC particularly implants if they have received training on comprehensive FP services in line with the Task Shifting and Task sharing policy.
**Tier 3 PPMVs (i.e. Health Qualified PPMVs**	1. These persons must fulfill the tier 1 eligibility criteria. 2. Must be Pharmacy technician	1. PPMV operators in this tier will be enabled to provide selected PHC services **(in line with the Task shifting and Task sharing policy**) 2. They will provide tier 1 services as well as use Rapid Diagnostic Test kits (RDTs), administer amoxicillin DT, conduct HIV Self Testing if trained 3. Sell self-injecting contraceptives and refer patients to PHCs and higher level facilities for nutrition counselling and treatment of any other common ailment

In addition to FP counselling training, CPs and Tier 2 and Tier 3 PPMVs received further three-day training on administration of injectable contraceptives and administration and removal of implant contraceptives. Providers practiced inserting implants on models and inserting injectables and implants on clients who voluntarily choose either of these two methods. CPs and PPMVs were referred to the social marketing sector to procure additional commodities. Supervisory monitoring visits were conducted within three months of the training to gauge compliance with protocols related to FP and infection prevention and control.

After the FP training, CPs and PPMVs were given training materials and BCS+ toolkit, which includes: a) BCS+ counseling cards; b) BCS+ algorithm; c) FP flip chart; and d) FP method brochures. In addition to the BCS+ toolkit, Tier 2 and Tier 3 PPMVs and CPs were given the Contraceptive Medical Eligibility Criteria (MEC) wheel check. An explanation of each tool is available in Table 2.

**Table 2:** Job Aids Given to Providers under IntegratE Project

Type of Job Aid	Description	Providers who received job aids
BCS+ counseling cards	It consists of information on pregnancy checklist, FP methods (use, side effects and duration), and information on HIV risk assessment, counseling, and testing	All providers
BCS+ Algorithm	Algorithm provides steps and questions to ask in the Pre-Choice Stage, Method Choice Stage, Post-Choice Stage. It also offers systematic screening of STIs /HIV including Prevention, Risk Assessment, and Counseling and Testing Stage.	All providers
FP Flip Chart	The FP Flip Chart contains information about FP methods (short-term, long-term, and permanent use), information on advantages and disadvantages for each method, possible side effects for each method, how to conduct counseling, HIV information, and pregnancy checklist.	All providers
FP method brochures	Pamphlets and leaflets on methods including their effectiveness, general information, how method works, important facts, side effects, how to use, and next steps.	All providers
MEC Wheel Check list	This wheel contains the medical eligibility criteria for starting use of contraceptive methods. It guides family planning providers in recommending safe and effective contraception methods for women with medical conditions or medically-relevant. Characteristics. The wheel includes recommendations on initiating use of nine common types of contraceptive methods.	Tier 2 PPMVs, Tier 3 PPMVs and CPs

## Methods

### Data

Data for this analysis come from an evaluation of the IntegratE Project, a pilot project that seeks to improve the quality of FP services delivered by CPs and PPMVs through a tiered accreditation system. Beginning in January 2019, 1465 CPs and PPMVs located in Kaduna and Lagos States were trained by the IntegratE project. In a quasi-experimental longitudinal design without a comparison group, 559 CPs and PPMVs completed a self-administered structured questionnaire to assess their knowledge related to the provision of FP counseling, and injectable and implant contraceptive services depending on their tier at three points in time: 1) before the training; 2) post/immediately after the training; and 3) 9-months after the training. The assessments were developed from established knowledge assessment tests used by the Project’s training partners.

Pre- and post/(immediately after the training) self-assessments were implemented in-person at the training venues from January to December 2019. Trained research assistants conducted the 9-month follow-up from March and May 2020. The research assistants were trained in study design, questionnaire content and research ethics approximately 2–3 weeks before data collection. Before the COVID-19 lockdown, research assistants visited providers’ premises and CPs/PPMVs completed the self-administered tool using Android tablets. During the lockdown, research assistants called CPs/PPMVs and read the questions and responses to respondents and then recorded their responses on the Android tablet.

All questionnaires asked the same knowledge questions on FP counselling, injectable and implants contraceptives (e.g. frequency and administration location, counseling on side effects, and eligibility criteria). The pre-test assessment also included questions on respondent characteristics and their cadre, state, experience providing injectable and implant contraceptives. The nine-month questionnaire included questions on FP services offered by CPs and PPMVs since the training, and their experience with the intervention including training received, job aids and monitoring visits. The results from the pre and posttest and 9 months follow up questionnaires were compared and analyzed in this paper.

All participants enrolled in the study were given detailed information about the study before consenting to participate. The study protocol was submitted and approved by the Population Council Institutional Review Board.

### Variables

#### Dependent variables

Three dependent variables were used in this paper: 1) general FP knowledge retention, 2) knowledge of injectable contraceptives retention, and 3) knowledge of implant contraceptives retention. General FP knowledge retention was assessed among Tier 1 and Tier 2 PPMVs and CPs, while knowledge of injectables and implant contraceptives retention were assessed only among Tier 2 PPMVs and CPs, as these tiers were trained in the provision of these methods. General knowledge and knowledge of injectable contraceptives categories each included 11 items, while 7 items were included in knowledge of implant contraceptives. Knowledge retention was measured for each item from posttest to the 9-month follow-up survey. Those who correctly answered a knowledge assessment question at posttest and answered it correctly at 9 months had the knowledge after the training and retained it over time. Respondents who did not know the item at posttest or at 9 months, who did not know it at posttest but did at 9 months, and who did not know it at posttest and at 9 months were not considered to have retained knowledge from posttest to 9-months.

Overall summary knowledge retention scores were developed for general FP knowledge, knowledge of injectable contraceptives, and knowledge of implant contraceptives. Scores were created for each of the three outcome variables separately. The total number of items that each respondent retained from posttest to 9 months was added. For general and knowledge of injectable contraceptives this is 11 items and for knowledge of implant contraceptives this is 7 items. Retention includes those who answered the question correctly at posttest and 9 months, while all other combinations are not counted as retention. For each outcome variable, the mean number of items that were retained was calculated. Those who scored above the mean were coded as 1 for high knowledge retention, and those who scored below the mean were coded as 0 for low knowledge retention. The mean scores were 5.7 out of 11 for general knowledge, 7.9 out of 11 for knowledge of injectable contraceptives, and 2.7 out of 7 for knowledge of implant contraceptives. Those who scored 6 or higher for general, 8 or higher for injectable, and 3 or higher for implant were considered to have high knowledge retention.

#### Independent variables

The key independent variable for this analysis were the five job aids given to all providers trained by the project. The job aids were categorized into three groups for their similarities: category 1 included BCS+, BCS+ algorithm, or FP flip charts (an alternative to BCS+), category 2 were the FP method brochures, and category 3 was the MEC wheel. For each of the three categories of job aids, responses were categorized as those who received the job aid during or after the training and those who did not receive the job aid.

Additional covariables considered were gender, age, tier, state, previous health facility experience, and receipt of specific job aids. Education was also considered but because it was used in the determination of tiers, it was highly correlation with tier and excluded from the analysis. For implant and knowledge of injectable contraceptives, a variable on whether the provider had offered these specific services in the 30 days preceding the interview was also included.

### Data analysis

Descriptive statistics were calculated for respondent characteristics at pretest and are presented by tier. Descriptive statistics for knowledge retention for general FP (among all providers), injectable (among Tier 2 PPMVs and CPs), and implant (among Tier 2 PPMVs and CPs) were conducted. The proportion who correctly answered each item at posttest was calculated, and among those who answered correctly at posttest, the proportion that retained the knowledge at 9 months is presented. The denominator for each item is dependent on the proportion who answered correctly at posttest.

Three multivariate logistic regression models were used to assess the effect of provider characteristics and receipt of job aids on general FP knowledge retention, knowledge of injectable contraceptives retention, and knowledge of implant contraceptives retention 9 months after the training. The outcome variable in each of the three models was high versus low knowledge retention according to the mean scores (as described above). The model for general knowledge was conducted among all respondents, while the implant and injectable models were conducted among Tier 2 PPMVs and CPs.

## Results

Table 3 presents background characteristics of providers included in the evaluation study by total and provider type. These data were collected before the FP training began. More females (60.3%) than males (39.2%) were included in the study. This was the case across all provider types. Most providers were between 30 and 49 years old (63.4%), and Tier 2 PPMVs were slightly younger than CPs and Tier 1 PPMVs. While a nearly even split of respondents came from Lagos and Kaduna overall, more CPs were enrolled in Lagos (72.8%) and more Tier 2 PPMVs were enrolled in Kaduna (93.0%). CPs (91.9%) and Tier 2 PPMVs (85.0%) were more likely to have ever worked in a health facility compared to Tier 1 PPMVs (45.9%). Slightly more than half (51.0%) of providers had never received any structured FP training and most who did, received it over a year before the intervention ago (35.4%).

**Table 3:** Background Characteristics of Private Providers in IntegratE Project Evaluation by Total and Provider Type, at Pretest

	CPs (***n*** = 272)	Tier 2 PPMVs (***n*** = 213)	Tier 1 PPMVs (***n*** = 74)	Total (***n*** = 559)
**Gender of Respondent**				
Male	47.8	30.0	33.8	39.2
Female	52.2	70.0	62.2	60.3
**Respondent Age**				
21–29	11.8	19.2	12.2	14.7
30–39	33.5	43.7	28.4	36.7
40–49	25.7	21.6	44.6	26.7
50+	24.6	14.1	14.9	19.3
**State**				
Lagos	72.8	7.0	51.4	44.9
Kaduna	27.2	93.0	48.6	55.1
**Ever worked in a health facility**				
No	4.8	15.0	50.0	14.7
Yes	91.9	85.0	45.9	83.2
**Last structured FP training**				
Never	54.0	47.9	48.6	51.0
Within the past month	0.4	0.9	4.1	1.1
2–6 months ago	2.6	4.7	12.2	4.7
7–12 months ago	4.0	7.0	2.7	5.0
Over 1 year ago	34.2	38.5	31.1	35.4

Table 4 shows retention of general FP counseling knowledge at posttest among all providers, and at 9-months follow-up but only for those providers who had the knowledge at posttest to assess knowledge retention. For many knowledge questions, provider knowledge was high at posttest and among those who responded correctly to the question at posttest, many were able to retain the information. For example, 98% of all providers correctly reported that condoms protect against pregnancy and HIV. Of those 98%, nearly all 99.5% continued to retain this information. Similarly, many providers (89.6%) at posttest knew the Intrauterine Device (IUD) was a modern method and most of them (87.4%) retained this information. In several instances, for example whether clients should be allowed to handle a sample of FP method during counseling, posttest knowledge was a little more than half (55.8%). But nearly all of those who had this knowledge at posttest retained it 9-months later (94.2%).

**Table 4:** Knowledge retention of FP from posttest to 9 months among CPs, Tier 1 PPMVs and Tier 2 PPMVs (*n* = 559)

	Posttest (***n*** = 559)	9-months later, among those that answered correctly at posttest
**Method that prevents both pregnancy and HIV**		(*n* = 548)
OCPs, implant, ECP (incorrect)	2.0	0.5
Condoms (correct)	98.0	99.5
**ECPs prevent pregnancy when taken up to 7 days**		(*n* = 323)
Incorrect	40.6	13.0
Correct	57.8	87.0
**SDM is a modern method**		(*n* = 137)
Incorrect	75.5	75.9
Correct	24.5	24.1
**LAM is a modern method**		(*n* = 151)
Incorrect	73.0	81.5
Correct	27.0	18.5
**IUD is a modern method**		(*n* = 501)
Incorrect	10.4	12.6
Correct	89.6	87.4
**Periodic abstinence is not a modern method**		(*n* = 502)
Incorrect	10.2	4.2
Correct	89.8	95.8
**Copper IUD is not a hormonal method**		(*n* = 270)
Incorrect	51.7	48.5
Correct	48.3	51.5
**A woman seeking FP selects her contraceptive method**		(*n* = 486)
Incorrect	13.1	6.8
Correct	86.9	93.2
**Clients should be allowed to handle a sample of FP method during counseling**		(*n* = 312)
Incorrect	44.2	5.8
Correct	55.8	94.2
**Clients should not be asked if over 18 years to assist them in choosing a method**		(*n* = 405)
Incorrect	25.8	15.1
Correct	72.5	84.9
**Client should go to the nearest health facility immediately and inform provider of method use, if there is a sudden change in health**		(*n* = 339)
Incorrect	39.4	49.0
Correct	60.6	51.0

For other knowledge measures, however, initial knowledge after the training was low and many of those who had the knowledge posttest lost it 9-months later. Only 27.0% knew that lactational amenorrhea method (LAM) is a modern method at posttest, and among them, only 18.5% retained this knowledge 9-months later. This was also the case for standard days method (SDM).

Table 5 shows knowledge retention of injectable contraceptive information including both Depo-Provera and Sayana Press. These questions were only asked among those trained in administration of injectable services - CPs and Tier 2 PPMVs. Overall knowledge was high for nine out of eleven questions at posttest, ranging from a low of 79.4% for knowledge of where Depo-Provera is injected to a high of 94.8% for the frequency of Depo-Provera injections. Retention of these nine questions remained for most who knew this information as posttest. Fewer had correct knowledge at posttest for two questions - the location of the Sayana Press injection (53.6%) and disposal of sharp box contents (42.3%). Most of those who knew where to dispose sharps at posttest retained the knowledge 9-months later (82.4%) while about half still knew where Sayana Press injection was given on the body 9-months later (51.9%).

**Table 5:** Knowledge of injectable contraceptives retention from posttest to 9 months among CPs and Tier 2 PPMVs (*n* = 485)

	Posttest (***n*** = 485)	9 months later, among those that answered correctly at posttest
**Syringe and needle are used to administer Depo-Provera**		(*n* = 420)
Incorrect	12.2	7.9
Correct	86.6	92.1
**Depo-Provera is administered intramuscularly**		(*n* = 451)
Incorrect	7.0	15.7
Correct	93.0	84.3
**Depo-Provera can be administered in buttocks, deltoid, thighs**		(*n* = 385)
Incorrect	20.6	19.5
Correct	79.4	80.5
**Depo-Provera is administered every 3 months**		(*n* = 460)
Incorrect	5.2	3.5
Correct	94.8	96.5
**Uniject is used to administer Sayana Press**		(*n* = 429)
Incorrect	8.7	4.9
Correct	88.5	95.1
**Sayana Press is administered subcutaneously**		(*n* = 441)
Incorrect	9.1	9.3
Correct	90.9	90.7
**Sayana Press can be administered on thighs, abdominal wall, back of the arm**		(*n* = 260)
Incorrect	44.7	48.1
Correct	53.6	51.9
**Sayana Press is administered every 3 months**		(*n* = 416)
Incorrect	14.2	9.6
Correct	85.8	90.4
**Depo Provera/Sayana Press have same active drug**		(*n* = 429)
Incorrect	11.5	4.2
Correct	88.5	95.8
**Sharp box contents disposed at health facility**		(*n* = 205)
Incorrect	57.7	17.6
Correct	42.3	82.4
**Injectables stored at room temperature and out of direct sunlight**		(*n* = 448)
Incorrect	4.7	7.1
Correct	92.4	92.9

Table 6 shows retention of knowledge of implant contraceptives 9-months after the posttest for CPs and Tier 2 PPMVs. Across the seven questions, knowledge was high at posttest for four questions and was retained by most providers 9-months later. Several knowledge questions were less known at posttest including the angle to insert the Implanon NXT needle (46.4%) and that implants may be difficult to remove when inserted under the fat (61.2%). For these two questions retention was lower than the other questions at 33.3 and 67.0%, respectively.

**Table 6:** Knowledge of implant contraceptives retention from posttest to 9 months among CPs and Tier 2 PPMVs (*n* = 485)

	Posttest (***n*** = 485)	9 months later, among those that answered correctly at posttest
**After Jadelle insertion, reusable instruments should be placed in a soapy water, wash and sterilize. (False**^a^**)**		(*n* = 105)
Incorrect	74.8	92.4
Correct	21.6	7.6
**Nexplanon Implant is effective for 3 years**		(*n* = 399)
Incorrect	17.7	25.3
Correct	82.3	74.7
**Implant is inserted under the skin of the arm**		(*n* = 465)
Incorrect	4.1	0.4
Correct	95.9	99.6
**After Implanon is inserted under the skin, verify the presence of the rod through gentle palpitation**		(*n* = 361)
Incorrect	21.6	22.7
Correct	74.4	77.3
**Implanon NXT needle is inserted at 30 degrees**		(*n* = 225)
Incorrect	53.6	66.7
Correct	46.4	33.3
**Implants inserted into fat under skins may be difficult to remove**		(*n* = 297)
Incorrect	38.8	33.0
Correct	61.2	67.0
**1st step to implant removal is palpate arm) and mark where the tip of the rod(s) are felt**		(*n* = 401)
Incorrect	12.8	24.2
Correct	82.7	75.8

^a^First step is to decontaminate in chlorine solution before this process

Table 7 shows the bivariate results of FP, injectable and implants knowledge retention 9-months later by jobs aids and respondent characteristics. Among providers who received BCS/algorithm/flipchart, 59.5% had knowledge of implant contraceptives 9-months later compared to 34.9% providers who did not receive these jobs aids (*p*-value = 0.00). Similarly, providers who received the MEC wheel were significantly more likely to have implant knowledge 9-months later than those who did not (60.5% compared to 42.2%, *p*-value = 0.00). Provider cadre was significantly associated with general FP knowledge retention where 77.2% of CPs compared to 41.3% of Tier 2 PPMVs and 33.8% of Tier 1 PPMVs retained knowledge 9-months later (*p*-value = 0.00). Knowledge of injectable contraceptives was significant among cadre where 71.4% of Tier 2 PPMVs compared to 58.1% of CPs retained implant knowledge (*p*-value =0.00).

**Table 7:** Bivariate results of FP, injectable and implement knowledge retention 9-months later by jobs aids and respondent characteristics

	FP knowledge retention		Knowledge of injectable contraceptives retention		Knowledge of implant contraceptives retention	
	%	n	%	n	%	n
**BCS+ counseling cards, BCS+ Algorithm or FP flip chart**						
Did not receive	59.6	52	58.1	43	34.9**	43
Received	57.6	507	64.5	442	59.5	442
**FP method brochures**						
Did not receive	59.7	288	62.2	233	62.2	233
Received	55.9	270	65.3	251	53.0	251
**MEC Wheel**						
Did not receive	52.3	151	57.8	83	42.2**	83
Received	59.8	408	65.2	402	60.5	402
**Gender**						
Male	58.0	219	55.7**	194	53.6	194
Female	58.2	337	69.4	291	59.8	291
**Age**						
21–29	57.3	82	64.4	73	63.0	73
30–39	56.1	205	60.3	184	57.6	184
40–49	54.4	149	69.0	116	59.5	116
50+	63.9	108	63.9	97	49.5	97
**Cadre**						
CP	77.2**	272	58.1**	272	57.7	272
Tier 2 PPMV	41.3	213	71.4	213	56.8	213
Tier 1 PPMV	33.8	74	–	–	–	
**State**						
Kaduna	50.0**	308	67.3	272	62.1	272
Lagos	67.3	251	59.6	213	51.2	213
**Ever worked in a Health Facility**						
No	30.5**	82	53.3	45	60.0	45
Yes	62.2	465	65.0	431	57.3	431
**Administered injectable in the last 30 days**						
No	–	–	55.0**	180	–	–
Yes	–	–	69.2	305	–	–
**Administered implant in the last 30 days**						
No	–	–	–	–	57.3	368
Yes	–	–	–	–	57.3	117

* *p*-value ≤ 0.05; ** *p*-value ≤ 0.01

Multivariate models are presented in Table 8 for all knowledge retention areas. For FP knowledge retention, providers who reported having the BCS+ counseling cards, BCS+ algorithm or the FP flip chart were more likely to retain knowledge on six or more questions (AOR: 2.92; 95% CI: 1.01–8.40; *p*- value = 0.05;). CPs and those who ever worked in a health facility were also more likely to retain knowledge 9-months later on six of the eleven knowledge questions.

**Table 8:** Adjusted odds ratios of FP, injectable and implement knowledge retention 9-months later

	FP knowledge retention (***n*** = 555)		Knowledge of injectable contraceptives retention (***n*** = 484)		Knowledge of implant contraceptives retention (***n*** = 484)	
	AOR	95% CI	AOR	95% CI	AOR	95% CI
**BCS+ counseling cards, BCS+ Algorithm or FP flip chart**						
Did not receive	ref		ref		ref	
Received	2.92*	1.01–8.40	1.01	0.32–3.21	1.14	0.36–3.60
**FP method brochures**						
Did not receive	ref		ref		ref	
Received	0.85	0.57–1.27	1.07	0.71–1.60	0.38**	0.25–0.57
**MEC Wheel**						
Did not receive	ref		ref		ref	
Received	1.20	0.63–2.27	2.13*	1.14–3.99	2.24*	1.17–4.28
**Gender**						
Male	ref		ref		ref	
Female	1.43	0.96–2.13	1.74**	1.17–2.59	1.48*	1.0–2.21
**Age**						
21–29	ref		ref		ref	
30–39	0.88	0.50–1.55	0.86	0.48–1.54	0.91	0.51–1.62
40–49	0.78	0.43–1.43	1.41	0.74–2.71	0.94	0.50–1.77
50+	1.04	0.54–2.02	1.15	0.59–2.25	0.72	0.37–1.40
**Cadre**						
CP	5.68**	3.25–9.92	0.66	0.37–1.19	1.90*	1.07–3.39
Tier 2 PPMV	ref		ref		ref	
Tier 1 PPMV	1.33	0.58–3.05	–	–	–	–
**State**						
Kaduna	ref		ref		ref	
Lagos	0.76	0.45–1.28	0.96	0.57–1.62	0.38**	0.22–0.65
**Ever worked in a Health Facility**						
No	ref		ref		ref	
Yes	2.22**	1.25–3.94	2.00*	1.04–3.87	0.88	0.45–1.71
**Administered injectable in the last 30 days**						
No	–	–	ref		–	–
Yes	–	–	1.48	0.94–2.33	–	–
**Administered implant in the last 30 days**						
No	–	–	–	–	ref	–
Yes	–	–	–	–	0.89	0.54–1.47

* *p*-value **≤**0.05; ** *p*-value **≤**0.01

In terms of knowledge of injectable contraceptives, CPs and Tier 2 PPMVs who reported receiving the MEC Wheel were 2.13 times more likely to retain knowledge of injectable contraceptives 9-months later on at least 6 knowledge of injectable contraceptives questions (AOR: 2.1; 95% CI: 1.14–3.99; *p*-value = 0.02). The odds of retaining injectable information were also more likely for females compared to males and for those who ever worked in health facility. Similar results were observed for knowledge of implant contraceptives retention except for ever worked in a health facility which was not statistically significant.

## Discussion

This study is one of the few studies of FP trainings to focus on both CPs and PPMVs’ FP knowledge retention. Unlike recent studies that examine knowledge retention with injectables training, this study also considers knowledge retention of implants. This study showed that nine months after the FP training, knowledge of key general FP, injectable and implant questions was retained. This suggests that CPs and PPMVs can be trained to provide a wide array of FP services. The findings further demonstrate that targeted training of CPs and PPMVs enhances FP knowledge. Results from this study are consistent with other studies in Nigeria that found positive impact of training on providers’ knowledge [10, 27]. This finding is promising for the strategy of task shifting task sharing in resource limited settings by expanding the role of CPs and PPMVs in FP service delivery in view of the major shortage of skilled providers for delivering FP services, especially for injectables and LARCs.

Another important finding from this study is that certain FP knowledge questions were better retained by CPs and PPMVs than other knowledge questions. For example, knowledge of implant contraceptives decreased 9 months later for two questions: the angle to insert the Implanon NXT needle and that an implant may be difficult to remove when inserted under the fat. Also, while this study focused on knowledge retention of providers who had the knowledge at posttest, it is important to note there were CPs and PPMVs who gained the information after posttest, suggesting that learning can be achieved through other implementation activities like monitoring and supervisory visits.

Overall, the results of this study showed that CPs and PPMVs retained knowledge of injectable contraceptives better than knowledge of implant contraceptives. Prior to the training, many of the CPs and PPMVs had already been providing injectables [33] without participating in any trainings or included in a supportive environment and even at posttest, the knowledge of injectable contraceptives scores were better than the knowledge of implant contraceptives scores. After trainings, providers have been offering injectable services more frequently than implant services; routine service data [35] showed that about 30 and 7% of women received injectables and implants, respectively. So, administering injectables over time may support providers’ retention of knowledge. To ensure method choice, however, additional support to providers is necessary so that they are offering full and complete information around implants and other FP methods. Results also suggest that trainings should focus on how and where to dispose sharps.

The study showed the importance of jobs aids for post training knowledge reinforcement for the providers. CPs and PPMVs who reported receiving job aids were more likely to answer the key knowledge questions correctly. This finding suggests that providing jobs aids to support CPs and PPMVs FP service provision can be effective in retaining knowledge over time. A similar study in Nigeria has shown that in addition to training, providing FP job aids was another strategy that can help PPMVs in effectively providing FP services over time [36]. FP Job aids supported healthcare workers with procedural, informational, or decisional “need-to-know” information in a simplified way [36]. Many studies in Africa have reported the usefulness of FP job aids. In Uganda and the Democratic Republic of Congo, [37] findings show that about half of the providers surveyed reported using FP checklists between 7 and 24 months after training. The providers who used these job aids indicated that they found them very useful. Another study conducted in South Africa reveals that a reinjection screening checklist aided the providers to excludes pregnancy for late arriving depot-medroxyprogesterone acetate (DMPA) clients and ultimately prevented unintentional discontinuation among those clients [38]. However, no difference was reported in terms of clients’ timeliness for reinjections. The multivariate results showed that among the FP jobs aids, the BCS cards and FP methods brochures helped CPS and PPMVs retained general FP, injectable and implants knowledge nine months after training. The CPs and PPMVs may likely or tend to use more the BCS cards and FP methods brochures in their day-to-day interactions with their clients. Providing FP jobs aids is one thing using the jobs aids is another thing. CPs and PPMVs must be trained on the use of jobs aids to reinforce their FP knowledge and enhance quality FP counselling and services.

This study also demonstrated that provider cadre was a significant factor in FP knowledge retention. General FP knowledge retention was more likely among CPs compared to PPMVs. Knowledge of implant contraceptives retention was more likely among CPs compared to PPMVs. This may be due to the fact the CPs have more years of prior training and exposure to health education and theoretical information in schools. Employing observational studies to assess the CPs and PPMVs FP competency and quality of care received by their clients may be a better way to compare these two providers.

We also found that having ever worked in health facilities was positively associated with retention of knowledge. General FP knowledge retention was more likely among CPs/PPMVs that have ever worked in a health facility compared to those that have not. Also, knowledge of injectable contraceptives retention was more likely among CPs/PPMVs that have ever worked in a health facility compared to those that have not. This result may be due to CPs and PPMVs exposure to training and practice in the public or private health facilities. CPs and PPMVs that have worked in health facilities were more likely to retain FP knowledge although this was not found statistically significant in a similar PPMV’s study in Nigeria [28].

Provision of an injectable and implant methods in the past 30 days was not found to be a statistically significant predictor of knowledge. Administering injection and implant within 1 month not sufficient to maintain knowledge among CPs and PPMVs. However, we need more studies that we look at the effect of client load or repeated practice on knowledge retention after training. Further, age, years of work and education attainment may not enhance retention of knowledge However, training program for CPs and PPMVs may be made more effective by taking into consideration gender, cadre, ever worked at health facilities.

## Limitations

There are a number of limitations to this study. The first was that our questions about FP knowledge only focused on general FP counselling, injectables and implants knowledge. We did not examine the CPs and PPMVs knowledge on FP methods side effect. The results may not reflect the comprehensive CPs and PPMVs knowledge of FP.

The second limitation is that COVID affected how data were collected in the follow-up interview where no in-person contact was allowed. In the wake of the COVID-19 pandemic research assistants administered the questionnaire over the phone. They read questions and responses to CPs and PPMVs before recording their responses on the Android tablet. This may have inherent flaws especially in the recording of responses and network problems. However, the research assistants were properly trained to ensure they had the necessary skills required for this data collection activity. Also, we endeavored to backcheck to validate the responses with randomly selected CPs and PPMVs during the data collection.

## Conclusion

The study has showed the likely impact of training on knowledge retention. The findings revealed that targeted training helped CPs and PPMVs retain the knowledge they were exposed to during training intervention. We can also conclude that apart from training, jobs aids were necessary for post training reinforcement and help CPs and PPMVs knowledge retention over time. Interventions need to take into consideration provision and training on FP job aids as one key strategy to help the CPs and PPMVs in providing FP services over time. However, to assess the value of task-sharing FP services to CPs and PPMVs over time, monitoring and supportive supervision are crucial [39]. Governments across the country need to integrate this cadre of health providers into their state ministry of health monitoring and supervision system to sustain provider competency and put a sustainable quality assurance mechanism.

## Data Availability

The dataset analyzed during the current study will be available on the Population Council’s Dataverse, https://dataverse.harvard.edu/dataset.xhtml?persistentId=doi:10.7910/DVN/AZNQ4J They will also be available from the corresponding author on reasonable request.

## Abbreviations

CP: Community Pharmacist
FP: Family planning
PPMV: Patent and proprietary medicine vendor
MEC: Medical Eligibility Criteria
BCS +: Balanced Counselling Strategy plus
OR: Odds ratio
PCN: Pharmacists Council of Nigeria
TSTS: Task-shifting and task-sharing
